# Postoperative Nausea and Vomiting According to Target-Controlled or Manual Remifentanil Infusion in Gynecological Patients Undergoing Pelviscopic Surgery: A Randomized Controlled Trial

**DOI:** 10.3390/jpm13020176

**Published:** 2023-01-19

**Authors:** Sangho Lee, Ann Hee You, Miyun Kim, Hee Yong Kang

**Affiliations:** Department of Anesthesiology and Pain Medicine, Kyung Hee University College of Medicine, Kyung Hee University Hospital, Seoul 02447, Republic of Korea

**Keywords:** postoperative nausea and vomiting, manual infusion, target-controlled infusion, remifentanil, pelviscopic surgery, emergence time

## Abstract

Background: We compared the incidence of postoperative nausea and vomiting (PONV) and postoperative outcomes, according to the remifentanil infusion method, during surgery in patients with a high-risk of PONV. Methods: Ninety patients undergoing elective gynecological pelviscopic surgery were randomly allocated to either target-controlled infusion (TCI, T) or manual (M) infusion. The primary outcome was the incidence of PONV until postoperative day (POD) 2. The secondary outcomes were perioperative heart rate (HR), blood pressure (BP), numerical rating scale pain scores up to POD2, and postoperative hospital length of stay. Results: Forty-four patients in the T group and 45 patients in the M group were analyzed. The total dose of remifentanil infusion was significantly higher in the T group (T group: 0.093 (0.078–0.112) μg/kg/min; M group: 0.062 (0.052–0.076) µg/kg/min, *p* < 0.001). Within POD2, the overall PONV was not significantly different (27 (61.4%) vs. 27 (60.0%), *p* = 0.895). The HR (82 ± 11.5/min vs. 87 ± 11.1/min, *p* = 0.046) and mean BP (83 ± 17.2 mmHg vs. 90 ± 16.7 mmHg, *p* = 0.035) were significantly lower in the T group after tracheal intubation. The other postoperative outcomes were comparable between the two groups. Conclusions: Although the total remifentanil infusion dose was higher in the T group than in the M group, the postoperative outcomes were similar. If stable vital signs are desired during tracheal intubation, remifentanil infusion with TCI should be considered.

## 1. Introduction

Postoperative nausea and vomiting (PONV) is a major cause of patient’s discomfort. This may impair the efficiency of distributing medical resources owing to a delay in transfer from the post-anesthetic care unit (PACU) to the general ward and the extension of hospital length of stay (LOS) [1,2]. In general, the incidence of PONV is 30–80% in patients with many risk factors [3,4]. In addition, unexpected hospitalizations may occur because of post-discharge nausea and vomiting (PDNV), which incurs additional medical costs. The risk factors for PONV include female sex, nonsmoking status, history of PONV, and postoperative use of opioids [5]. The risk factors for PDNV include female sex, history of PONV, age < 50 years, use of opioids in the PACU, and nausea in the PACU [6]. The risk factors related to anesthesia include the use of nitric oxide, inhaled anesthetics, opioids during surgery, and extended anesthesia time. PONV may be more prevalent in strabismus surgery, neurosurgery, laparoscopic surgery, abdominal surgery, and gynecological surgery [7,8]. Various interventions, such as antiemetic and steroids use, aroma therapy, and acupuncture, are being employed in clinical practice to reduce PONV in the high-risk patient group [2,9,10].

Opioids are commonly used for sedation and pain control in patients, depending on the surgical procedure. However, when opioids are used, three sensory pathways are stimulated: the vestibular nuclei, area postrema, and vagal afferent fibers. These stimuli cause a vomiting reflex in the local brainstem areas through the nucleus of the solitary tract, and projection to the mid- and forebrain for the perception of nausea [11,12]. Remifentanil has a short half-life; therefore, the level of stimulation is easy to control in each surgical scenario. In addition, it is mainly used for continuous intravenous infusion during surgery because it has a short context-sensitive half-life and relatively quick recovery time compared to other opioids [13,14]. In the case of the continuous infusion of remifentanil, two methods are available: a manual infusion method that determines the injection rate per hour, and a target-controlled infusion (TCI) method, which uses a program that calculates the target concentration in advance using a pharmacological formula [15]. Manual infusion can be intuitively used by physicians, which is among its advantages. Conversely, in the TCI method, a bolus dose is automatically infused by simultaneously calculating the plasma concentration (Cp) and the effect site (Ce). Subsequently, when Ce reaches the set level, it is infused at a constant rate. This makes it possible to quickly and easily control the desired pharmacological effects [16]. These two infusion methods are commonly used in clinical practice.

However, to our knowledge, no studies have assessed the difference in the incidence of PONV according to TCI or manual infusion of remifentanil alone. Therefore, this study aimed to evaluate the incidence of PONV according to the infusion method of remifentanil in gynecological patients undergoing pelviscopic surgery who are at a high-risk of PONV. We hypothesized that remifentanil administered by TCI is associated with a lower incidence when compared with manual infusion.

## 2. Materials and Methods

### 2.1. Study Design and Ethics

This study was designed to evaluate PONV based on the infusion method of remifentanil during gynecological pelvic surgery in an assessor-blinded parallel-group randomized controlled trial. Ethical approval was obtained from the Institutional Review Board of Kyung Hee University Hospital (KHUH 2022-05-083-001) on 28 June 2022. The trial was conducted in compliance with the Declaration of Helsinki and registered in the Clinical Research Information Service (No. KCT0007703; registration date: 1 July 2022; principal investigator: Hee Yong Kang). The study protocol was available from the Clinical Research Information Service. Written informed consent was obtained from all the participants. The study report complied with the Consolidated Standards of Reporting Trials checklist.

### 2.2. Participants

Adult women aged 19–69 years who were scheduled for elective gynecological pelviscopic surgery at Kyung Hee University Hospital were eligible for trial inclusion. The exclusion criteria were pregnancy, allergy to any study drug, antiemetics administered within 24 h preoperatively, severe obesity with a body mass index of 35 kg/m^2^ or higher, and American Society of Anesthesiologists class III or higher. Recruitment began in July 2022 and ended in November 2022.

### 2.3. Outcomes

The primary outcome was the incidence of PONV within 48 h after surgery. PONV was evaluated through patient interviews and medical record reviews at the PACU, postoperative days (POD) 1, and POD2. It was defined as the time at which the patient complained of nausea or vomiting, or when antiemetic drugs were administered.

The secondary outcomes included numerical rating scale (NRS) pain scores in the PACU, POD1, and POD2. Perioperative blood pressure (BP), heart rate (HR), emergence time to eye opening, tracheal extubation, and exit from the operating room after desflurane cessation were evaluated. The number of patients who were administered opioids or analgesics in the PACU, POD1, and POD2 were recorded. Additionally, the PACU LOS and postoperative hospital LOS were recorded.

### 2.4. Randomization and Masking

The patients were randomly assigned to the intervention (TCI, T) or control (manual, M) group using sealed opaque envelopes on the morning of surgery. A computer-generated random allocation sequence was created using Excel 2019 (Microsoft) with a 1:1 allocation and random block sizes. The study participants and outcome assessors were blinded to the group allocation, whereas the anesthesia providers were not because of a significant difference in the method of drug infusion.

### 2.5. Procedures

After entry to the operating room, HR, BP, and peripheral oxygen saturation were recorded for all patients; subsequently, dexamethasone 0.1 mg/kg and glycopyrrolate 0.005 mg/kg were administered. After pre-oxygenation, propofol 2 mg/kg and rocuronium 0.8 mg/kg were administered. The anesthesia was maintained with desflurane and was adjusted according to the bispectral index (BIS) level goal, which is from 30 to 60.

In the case of the T group, the Ce of remifentanil before intubation was set to 5.0–7.0 ng/mL using the syringe pump with a Minto model (Orchestra^®^ Base Primea, Fresenius Kabi, VL, France) [17]. During surgery, the patients’ BP and HR were monitored, and the Ce of remifentanil was adjusted within the range of 1.0–3.0 ng/mL. In the M group, 0.7–1 µg/kg of remifentanil was administered before intubation using an infusion pump (Terufusion^®^ Infusion Pump, Terumo, Tokyo, Japan). During surgery, remifentanil was titrated within the range of 0.05–0.2 µg/min/kg against HR and BP within a 20% range of baseline measurements. In both groups, if vital signs could not be controlled by remifentanil alone, a vasoconstrictor (phenylephrine, 50 µg) or vasodilator (nicardipine, 500 µg) was administered.

Remifentanil was discontinued during skin closure, and desflurane was discontinued when the position was changed from lithotomy to supine after surgery, and glycopyrrolate 0.005 mg/kg and pyridostigmine 0.25 mg/kg were administered as reversal agents of neuromuscular blockers. At the end of surgery, fentanyl-based (17.5 µg/kg) intravenous patient-controlled analgesia was administered to all of the participants until POD2. Additional opioids or analgesics were administered when patients complained of pain with an NRS pain score of ≥3 or if the patient required analgesics. In the PACU, fentanyl was administered as a bolus dose of 1 µg/kg. On POD1, intravenous acetaminophen 750 mg and per os ibuprofen 200 mg were used, and on POD2, ibuprofen was administered alone.

### 2.6. Outcome Assessments and Data Collection

Based on the medical records, the overall incidence of PONV was evaluated from PACU to POD2.

The participants’ HR and BP were recorded at the initial visit and before and after tracheal intubation, skin incision, carbon dioxide insufflation, specimen removal, and skin closure stage. The total amount of remifentanil and other drugs administered during the surgery was recorded. The duration of emergence from desflurane cessation to eye opening, tracheal extubation, and exit from the operating room was recorded.

After leaving the operating room, the participants’ HR and BP were measured in the PACU. The incidence of PONV was recorded in the PACU, POD1, and POD2. Additionally, the opioid and analgesic requirements in the PACU, POD1 and POD2, PACU LOS, and postoperative hospital LOS were also evaluated.

### 2.7. Sample Size Calculation

In a previous study comparing both propofol and remifentanil in patients who underwent mastoidectomy surgery, in T and M groups, the incidence of vomiting in the PACU was 9% and 33%, respectively [15]. As a result of the G-power analysis using these data (exact test, proportions: inequality, two independent groups (Fisher’s exact test); a priori: compute required sample size—given α, power, effect size, two tails, proportion p1 0.09, proportion p2 0.33, α err 0.1, power 0.8, and allocation ratio N2/N1 1), 40 participants in each group were calculated. Considering a dropout rate of 10%, the total calculated target number of participants was 90, with 45 patients in each group.

### 2.8. Statistical Analysis

The data are presented as medians (interquartile ranges) or numbers (%). The categorical variables were analyzed using the Fisher’s exact test or chi-square test. The normality of continuous variables was evaluated using the Shapiro–Wilk test. Independent variable t-tests or Wilcoxon rank-sum tests were used for continuous variables. Two-sided *p*-values < 0.05 were considered statistically significant. The statistical analyses were performed using SPSS (version 22.0; SPSS Inc., Chicago, IL, USA).

## 3. Results

### 3.1. Study Population and Intraoperative Data

**Figure 1:**
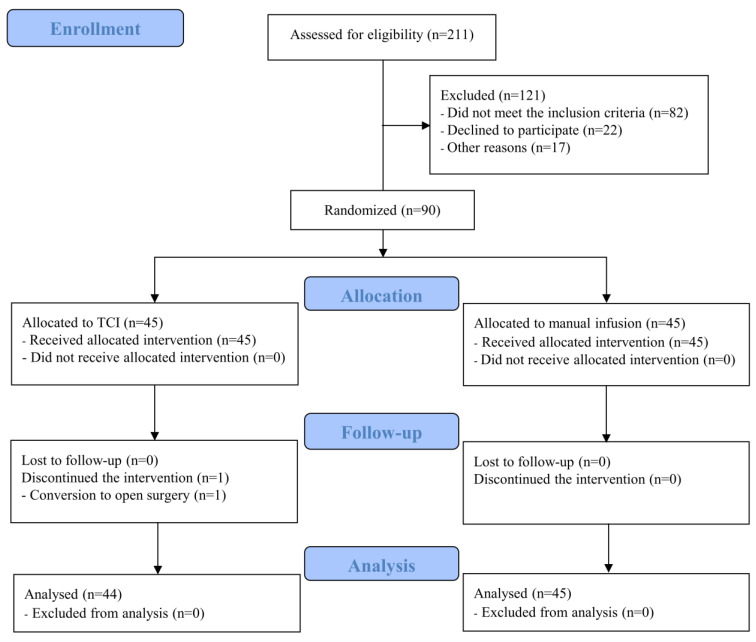
CONSORT diagram of the patient enrollment. TCI, target-controlled infusion; CONSORT, Consolidated Standards of Reporting Trials.

A total of 211 patients met the eligibility criteria: 90 patients were randomly assigned and 121 patients were excluded. Forty-five patients were equally assigned to both groups; one patient dropped out in the T group because of conversion from pelviscopic surgery to laparotomy. Finally, 44 and 45 patients in the T and M groups, respectively, were analyzed (Figure 1).

There were no statistically significant differences in the patients’ demographics between the two groups. The type of surgery, total intraoperative fluid administered, estimated blood loss, urine output, and operation time were comparable between the groups. However, the median dose of remifentanil infusion during surgery was 0.093 (0.078–0.112) µg/kg/min in the T group, which was significantly higher than the median dose of 0.062 (0.052–0.076) µg/kg/min in the M group (*p* < 0.001) (Table 1).

### 3.2. Primary Outcome

**Figure 2:**
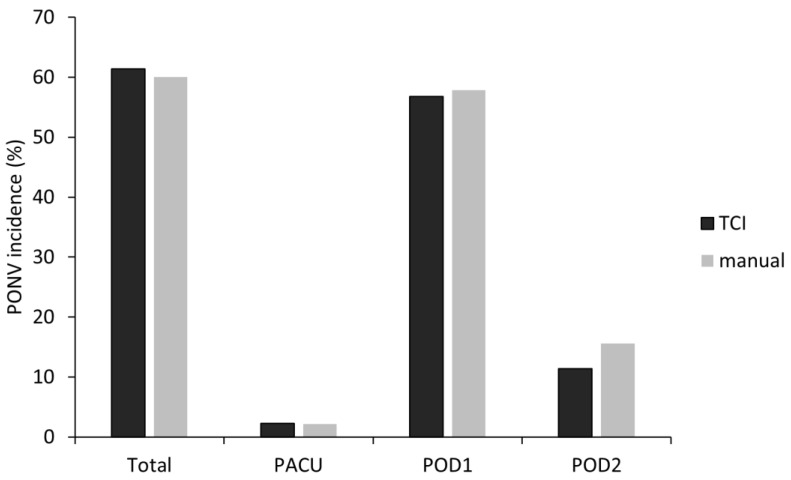
PONV incidence in total and different period. PONV, postoperative nausea and vomiting; TCI, target-controlled infusion; PACU, post-anesthetic care unit; POD, postoperative day.

Within POD2, the overall incidence of PONV was 27 (61.4%) in the T group and 27 (60.0%) in the M group, with no statistically significant difference between the groups (*p* = 0.895) (Figure 2).

In the PACU, PONV occurred in one patient in both groups, and the incidence was comparable (2.3% in the T group vs. 2.2% in the M group, *p* = 0.987). In POD1, PONV occurred in 25 (56.8%) patients in the T group and 26 (57.8%) patients in the M group (*p* = 0.927), and in POD2, five (11.4%) patients in the T group and seven (15.6%) patients in the M group (*p* = 0.563); however, the difference was not statistically significant (Figure 2).

### 3.3. Secondary Outcomes

**Figure 3:**
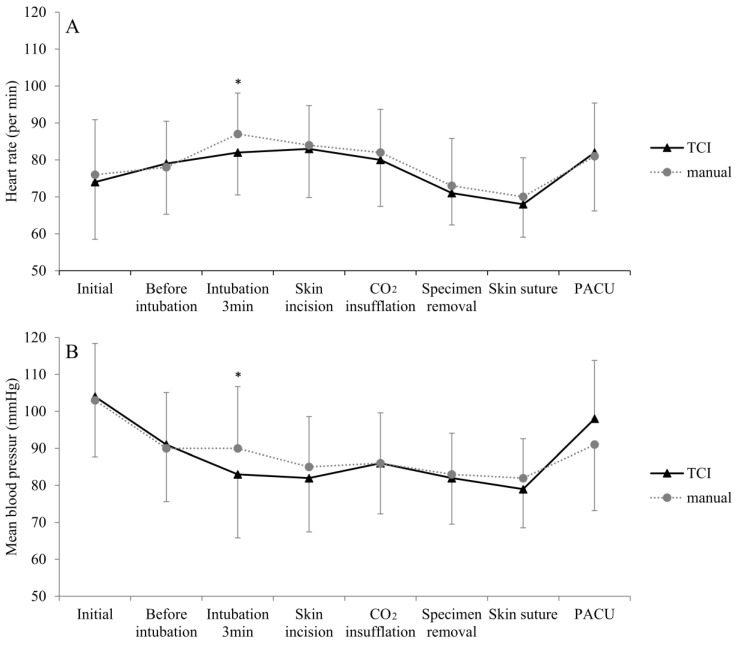
Trend in vital signs by different perioperative stages. (**A**) HR. (**B**) mBP. Data denote means ± standard deviations. * Statistical significance. HR, heart rate; mBP, mean blood pressure; TCI, target-controlled infusion; CO_2_, carbon dioxide; PACU, post-anesthetic care unit.

The HR was significantly lower in the T group than in the M group 3 min after intubation (T group: 82 ± 11.5/min vs. M group: 87 ± 11.1/min, *p* = 0.046) (Figure 3A). With the exception of 3 min after tracheal intubation, the perioperative HR was similar in both groups (Supplementary Table S1). There was no statistically significant difference in the perioperative mean BP (mBP) between the two groups, except at 3 min after intubation. At 3 min after intubation, the mBP was significantly lower in the T group than in the M group (83 ± 17.2 mmHg in the T group vs. 90 ± 16.7 mmHg in the M group, *p* = 0.035) (Figure 3B). The mBP in PACU was 98 ± 15.8 mmHg in the T group, which was higher than that of the M group (91 ± 17.8 mmHg), but the difference was not statistically significant (*p* = 0.05) (Supplementary Table S2).

At the time of emergence, there was no significant difference between the two groups in terms of eye opening, extubation, or exit from the operating room. The number of patients who were administered opioids or analgesics in the PACU, POD1, and POD2 were similar in both groups. The NRS pain scores in the PACU, POD1, POD2, PACU LOS, and postoperative hospital LOS were comparable between the two groups (Table 2).

## 4. Discussion

This prospective randomized clinical trial compared the incidence of PONV according to the manual or TCI infusion of remifentanil in patients undergoing gynecological pelviscopic surgery. The total intraoperative amount of remifentanil was significantly higher in the T group; however, there was no statistically significant difference in the incidence of PONV between the two groups. The BP and HR after tracheal intubation were significantly lower in the T group than in the M group. The other perioperative period BP, HR, emergence time, PACU LOS, postoperative NRS pain scores, and postoperative hospital LOS were comparable between groups.

According to Hozumi et al., the dose of remifentanil administered (>0.2 µg/kg/min) during elective mastectomy under general anesthesia was an independent risk factor for PONV [18]. In our study, the median intraoperative dose of remifentanil was ≤0.1 µg/kg/min for both groups; consequently, there may be no significant difference in the incidence of PONV.

Previous studies comparing TCI and manual infusion as remifentanil injection methods reported that the total dose of remifentanil was higher in the manual infusion group [15,19]. In our study, the higher dose of remifentanil infusion during surgery in the T group may have resulted from the large amount of remifentanil administered immediately before tracheal intubation. When using the TCI system, increasing the Ce setting requires a higher Cp level and a bolus dose is administered simultaneously [17]. The amount of remifentanil infusion in the T group at this stage was greater than that in the M group, which may have caused a difference in the total amount of remifentanil and the lower BP and HR in the T group after tracheal intubation. Although there was no significant difference, except for the stage of tracheal intubation, the overall intraoperative BP and HR tended to be lower in the T group, which implies that the dose of remifentanil was higher in the T group during surgery. However, because the postoperative outcomes were similar in both groups, the difference in the amount of remifentanil infusion was considered clinically irrelevant. For stable vital signs during the tracheal intubation stage, the use of TCI may be considered.

The BP and opioid requirement of the PACU are also noteworthy. Although there was no statistically significant difference between the two groups, in the T group, the mBP was approximately 7.1 mmHg higher than that in the M group (Supplementary Table S2), and the number of opioid-administered patients was more than twice that of the T group (Table 2). Clinically, the BP and NRS pain scores were not sufficiently high to be considered, but it can be considered when weak hyperalgesia occurs due to the large amount of remifentanil administered [20,21]. In the case of TCI, the estimated time required to decrease to 1 ng/mL was displayed on the built-in program, and by reducing the infusion rate of remifentanil by referring to this at the end of surgery, there is a possibility that it may be removed from the body slightly earlier than in the M group. The statistical significance of this finding must be confirmed in a larger study.

### Limitations

This study has several limitations. First, double-blinding could not be performed because of the differences between the TCI and manual infusion methods.

Second, intraoperative remifentanil was titrated against BP and HR. In studies related to anesthetic agents, such as propofol, the infusion dose was adjusted objectively by monitoring the depth of anesthesia, such as the BIS or patient state index [22,23]. In the case of remifentanil, pain monitoring devices such as the surgical pleth index or analgesia nociception index are not widely used; therefore, there are limitations to titrating against them [24,25]. In the future, if the dose of remifentanil is adjusted using pain monitoring equipment, an objective and highly reproducible study can be performed. Additionally, the intraoperative dose of remifentanil was smaller than in previous studies [15,19,22]. However, the dose of remifentanil for maintaining general anesthesia is suggested as 0.05–0.2 µg/min/kg, and the dose in our study also corresponds to this range. The intraoperative BP and HR were maintained at appropriate levels, even at these doses. Currently, when there is a trend to reduce opioid use, the significance of this study can be increased by using a low-dose opioid.

Finally, the patients were limited to those undergoing gynecological pelviscopic surgery, and the number of participants was relatively small. However, female patients, pelviscopic surgery, and intraoperative opioid use may increase the incidence of PONV [26,27]. The strength of this study is that it was conducted in patients expected to have a high incidence of PONV. This study is a part of the efforts to reduce PONV in high-risk patients and is expected to decrease the additional medical costs incurred due to PONV. Additionally, although the number of participants was relatively small, the randomization was sufficient, and there were no significant differences in the demographic data between the two groups. Therefore, this study was sufficient to only compare different remifentanil infusion methods.

## 5. Conclusions

These two remifentanil infusion methods may be reasonably used in terms of the incidence of PONV and postoperative complications in patients with a high-risk of PONV. However, the HR and BP tended to be lower in the T group only after tracheal intubation. If stable vital signs are desired during tracheal intubation, remifentanil infusion with TCI may be considered. Further studies with larger and more diverse patient populations are warranted.

## Figures and Tables

**Table 1:** Patient characteristics and intraoperative data of the study cohort.

	TCI (n = 44)	Manual (n = 45)	*p* Value
Demographics			
Age (y)	43 (35–52)	47 (40–52)	0.684
Height (cm)	160 (154–165)	160 (155–163)	0.742
Weight (kg)	60 (52.5–72.5)	58 (53.8–61.4)	0.308
Body mass index (kg/m^2^)	23.6 (20.8–28.2)	23.1 (20.9–25.2)	0.331
ASA class (I/II)	27 (61.4%)/17 (38.6%)	27 (60.0%)/18 (40.0%)	1.000
PONV risk score			0.747
2	4 (9.1%)	3 (6.7%)	
3	22 (50.0%)	26 (57.8%)	
4	18 (40.9%)	16 (35.6%)	
Diabetes	5 (11.4%)	2 (4.4%)	0.413
Hypertension	5 (11.4%)	7 (15.6%)	0.788
Hemoglobin (g/dL)	13.4 (12.4–14.0)	12.8 (12.0–13.3)	0.092
Platelet (×10^3^/μL)	268 (236–321)	273 (259–326)	0.416
Albumin (g/dL)	4.5 (4.3–4.6)	4.3 (4.2–4.6)	0.137
Total bilirubin (mg/dL)	0.6 (0.4–0.7)	0.6 (0.5–0.8)	0.329
Creatinine (mg/dL)	0.7 (0.6–0.7)	0.6 (0.6–0.7)	0.483
Intraoperative data			
Remifentanil total (μg)	350 (255–499)	230 (155–330)	0.001 *
Remifentanil infusion rate (μg/kg/min)	0.093 (0.078–0.112)	0.062 (0.052–0.076)	<0.001 *
Anesthesia time (min)	100 (75–118)	100 (75–130)	0.752
Surgical time (min)	70 (46–88)	65 (45–95)	0.831
Crystalloid infusion (mL)	300 (225–400)	400 (200–500)	0.192
Estimated blood loss (mL)	65 (50–150)	100 (50–150)	0.412
Urine output (mL)	100 (50–150)	150 (50–200)	0.108
Type of surgery			0.832
Hysterectomy	22 (50.0%)	21 (46.7%)	
Adnexectomy	21 (47.7%)	22 (48.9%)	
Myoma enucleation	1 (2.3%)	2 (4.4%)	

Data are presented as medians (interquartile ranges) or numbers (%). * Statistical significance. TCI, target–controlled infusion; ASA, American Society of Anesthesiologists; PONV, postoperative nausea and vomiting.

**Table 2:** Postoperative secondary outcomes.

	TCI (n = 44)	Manual (n = 45)	*p* Value
Emergence time			
Eye open (s)	265 (225–335)	289 (234–342)	0.266
Extubation (s)	327 (248–395)	324 (270–393)	0.485
Exit the OR (s)	366 (282–435)	361 (300–435)	0.752
PACU LOS (min)	30 (30–30)	30 (30–35)	0.118
PACU opioid requirement (n)	10 (22.7%)	5 (11.1%)	0.238
POD1 analgesics requirement (n)	29 (65.9%)	34 (75.6%)	0.443
POD2 analgesics requirement (n)	19 (43.2%)	24 (53.3%)	0.456
NRS pain scores			
PACU	3 (3–3)	3 (3–3)	0.139
POD1	3 (2–3)	3 (3–3)	0.088
POD2	2 (2–3)	2 (2–3)	0.308
Postoperative hospital LOS (d)	4 (3–4)	4 (3–4)	0.372

Data are presented as medians (interquartile ranges) or numbers (%). TCI, target–controlled infusion; PACU, post-anesthetic care unit; LOS, length of stay; NRS, numerical rating scale; POD, postoperative day.

## Data Availability

The datasets analyzed during the study are available from the corresponding author upon reasonable request.

## Supplementary Materials

The following supporting information can be downloaded at: https://www.mdpi.com/article/10.3390/jpm13020176/s1, Table S1: Perioperative heart rate at different stages; Table S2: Perioperative blood pressure at different stages.
