# Supplementary material for: Postoperative Nausea and Vomiting According to Target-Controlled or Manual Remifentanil Infusion in Gynecological Patients Undergoing Pelviscopic Surgery: A Randomized Controlled Trial
Source: J Pers Med. 2023 Jan 19;13(2):176. doi: 10.3390/jpm13020176 (PMC9966727; doi:10.3390/jpm13020176)
Supplement: Supplementary file 1 [file jpm-13-00176-s001.zip › jpm-2119500-supplementary.pdf]

Table S1. Perioperative heart rate at different stages.

	TCI	Manual	P-value
Initial	73.7 ± 15.5	75.7 ± 14.9	0.539
Before intubation	79.2 ± 11.5	78.2 ± 12.7	0.709
3 min after intubation	81.7 ± 11.5	86.5 ± 11.1	0.046*
Skin incision	83.3 ± 13.2	83.6 ± 10.7	0.898
CO_2 insufflation	79.5 ± 12.6	81.6 ± 11.7	0.417
Specimen removal	71.2 ± 8.6	72.8 ± 12.8	0.490
Skin suture	68.0 ± 8.9	69.8 ± 10.6	0.393
PACU	82.2 ± 13.4	81.0 ± 14.8	0.683

Data denote means ± standard deviations. * Statistical significance. TCI, target-controlled infusion; CO_2, carbon dioxide; PACU, post-anesthetic care unit.

Table S2. Perioperative blood pressure at different stages.

	TCI	Manual	P-value
Systolic blood pressure			
Initial	140.7 ± 21.0	140.2 ± 21.7	0.915
Before intubation	122.2 ± 16.7	121.0 ± 17.1	0.737
3 min after intubation	114.4 ± 21.4	119.0 ± 18.1	0.275
Skin incision	109.5 ± 21.4	113.2 ± 13.9	0.344
CO_2 insufflation	115.3 ± 16.1	113.7 ± 16.5	0.657
Specimen removal	111.0 ± 12.3	110.7 ± 13.2	0.908
Skin suture	108.0 ± 11.3	109.1 ± 10.9	0.625
PACU	133.2 ± 22.4	124.3 ± 21.6	0.058
Mean blood pressure			
Initial	104.4 ± 14.4	102.5 ± 15.3	0.558
Before intubation	91.4 ± 14.1	89.9 ± 14.4	0.606
3 min after intubation	82.5 ± 17.2	90.2 ± 16.7	0.035*
Skin incision	81.8 ± 14.6	85.3 ± 13.6	0.249
CO_2 insufflation	86.0 ± 13.7	86.0 ± 13.6	0.994
Specimen removal	81.6 ± 12.5	83.4 ± 11.1	0.477
Skin suture	79.2 ± 10.5	81.5 ± 10.6	0.299
PACU	97.6 ± 15.8	90.5 ± 17.8	0.050
Diastolic blood pressure			
Initial	87.2 ± 12.8	85.4 ± 12.0	0.499
Before intubation	78.2 ± 12.8	75.3 ± 13.0	0.285
3 min after intubation	69.4 ± 15.4	78.3 ± 16.6	0.010*
Skin incision	70.1 ± 15.7	74.3 ± 14.1	0.182
CO_2 insufflation	74.2 ± 12.4	75.5 ± 14.5	0.643
Specimen removal	70.1 ± 13.2	71.5 ± 11.1	0.590
Skin suture	66.7 ± 10.9	69.6 ± 9.9	0.189
PACU	79.4 ± 15.0	73.1 ± 17.1	0.069

Data denote means ± standard deviations. * Statistical significance. TCI, target-controlled infusion; CO_2, carbon dioxide; PACU, post-anesthetic care unit.
